# ResistanceSim: development and acceptability study of a serious game to improve understanding of insecticide resistance management in vector control programmes

**DOI:** 10.1186/s12936-018-2572-2

**Published:** 2018-11-13

**Authors:** Edward K. Thomsen, Charlotte Hemingway, Andy South, Kirsten A. Duda, Claire Dormann, Robert Farmer, Michael Coleman, Marlize Coleman

**Affiliations:** 1 Liverpool School of Tropical Medicine, Pembroke Place, Liverpool, L3 5QA UK; 2 Extra Mile Studios, 142 West Nile St, Glasgow, G1 2RQ UK; 3 Present Address: IVCC, Pembroke Pl, Liverpool, L3 5QA UK

**Keywords:** Serious games, Insecticide resistance management, Insecticide resistance, Vector control, Capacity building, Training

## Abstract

The use of insecticides is the cornerstone of effective malaria vector control. However, the last two decades has seen the ubiquitous use of insecticides, predominantly pyrethroids, causing widespread insecticide resistance and compromising the effectiveness of vector control. Considerable efforts to develop new active ingredients and interventions are underway. However, it is essential to deploy strategies to mitigate the impact of insecticide resistance now, both to maintain the efficacy of currently available tools as well as to ensure the sustainability of new tools as they come to market. Although the World Health Organization disseminated best practice guidelines for insecticide resistance management (IRM), Rollback Malaria’s Vector Control Working Group identified the lack of practical knowledge of IRM as the primary gap in the translation of evidence into policy. ResistanceSim is a capacity strengthening tool designed to address this gap. The development process involved frequent stakeholder consultation, including two separate workshops. These workshops defined the learning objectives, target audience, and the role of mathematical models in the game. Software development phases were interspersed with frequent user testing, resulting in an iterative design process. User feedback was evaluated via questionnaires with Likert-scale and open-ended questions. The game was regularly evaluated by subject-area experts through meetings of an external advisory panel. Through these processes, a series of learning domains were identified and a set of specific learning objectives for each domain were defined to be communicated to vector control programme personnel. A simple “game model” was proposed that produces realistic outputs based on player strategy and also runs in real-time. Early testing sessions revealed numerous usability issues that prevented adequate player engagement. After extensive revisions, later testing sessions indicated that the tool would be a valuable addition to IRM training.

## Background

In 2000, with the signing of the Abuja Declaration, leaders from malaria-endemic countries across sub-Saharan Africa committed themselves to decrease the burden of malaria [1]. This increase in political will was rapidly followed by greater financial support from global partners. As a result, after three decades of stagnation since the close of the World Health Organization (WHO) Global Malaria Elimination Programme in 1969, the last 18 years has seen a rapid scale-up of malaria control interventions. Insecticide-based vector control lies at the heart of the global strategy.

Pyrethroids, with their low mammalian toxicity, long residual life, and relatively low production cost, became the dominant insecticide class of choice during the scale-up. At the time of the declaration, resistance to pyrethroids was almost negligible, with just a few populations of vectors exhibiting resistance on the African continent [2]. Now, with the extensive use of these insecticides for both insecticide-treated nets (ITNs) and indoor residual spraying (IRS), not a single country in sub-Saharan Africa is free from pyrethroid resistance [3, 4]. Resistance to all other classes of public health insecticides is ubiquitous as well. Consequently, the ability to control the vectors responsible for transmitting the disease is compromised.

The path to this situation is characterized by an insufficient safeguarding of the available insecticide products. Despite proven strategies to curb resistance [5], vector control programmes around the world have relied exclusively on monotherapies, mainly pyrethroids, for years. Cross-resistance between this and other insecticide classes limits the number of alternatives, resulting in development of further resistance to these products as well. These practices result in some countries without viable vector control strategies.

If there is not a culture change surrounding public health insecticide use, there is a risk that the effectiveness of existing and new insecticides will be compromised by resistance. Recognizing the gravity of the current situation, the WHO published the Global Plan for Insecticide Resistance Management (GPIRM) [6], which provides technical recommendations for national control and elimination programmes to sustainably manage resistance. However, the operational implementation of these recommendations is lacking, and innovative solutions are required to communicate the principles and implications of insecticide resistance management (IRM).

‘Serious games’ are games designed for purposes beyond mere entertainment. They blend the engaging, fun, and challenging components of gaming with the goal of supplying the player with skills and knowledge useful in real-life situations, ultimately supporting attitude and behaviour change. Modern instructional design theory suggests that effective learning is accomplished through active involvement of the learner, a self-directed approach, and working with realistic scenarios [7]. All of these criteria are central to a simulation game. In addition, social cognitive theory is based on the idea that behaviour is driven by the understanding of the world in which a person lives, including the positive and negative outcomes witnessed as a result of choices made [8] and beliefs in personal efficacy; games influence the player’s understanding of the world around them by enabling them to explore complex problems in a safe setting, allowing them to make mistakes and learn from them without real-world consequences.

The value of serious games has seen increased attention from many industries since 2002 [9], most notably the healthcare sector. Games have been used to improve adherence to self-medication among cancer patients [10], relieve symptoms of depression [11], and train medical and surgical personnel [12], among many other applications. However, games have not yet been used among the implementers of public health programmes, where relatively few individuals are responsible for engaging in complex decision-making processes that ultimately impact the health of tens of thousands of people. In addition, there have been relatively few applications of serious games in low and middle income countries where increased computer literacy is producing a generation that may be particularly receptive to digital gaming solutions.

Here a serious game was developed to improve understanding and adoption of strategies to manage insecticide resistance among vector control programmes in malaria-endemic countries. Here, the game development and the process of developing it, is presented with results from preliminary acceptability studies.

## ResistanceSim

### Open simulation

**Fig. 1:**
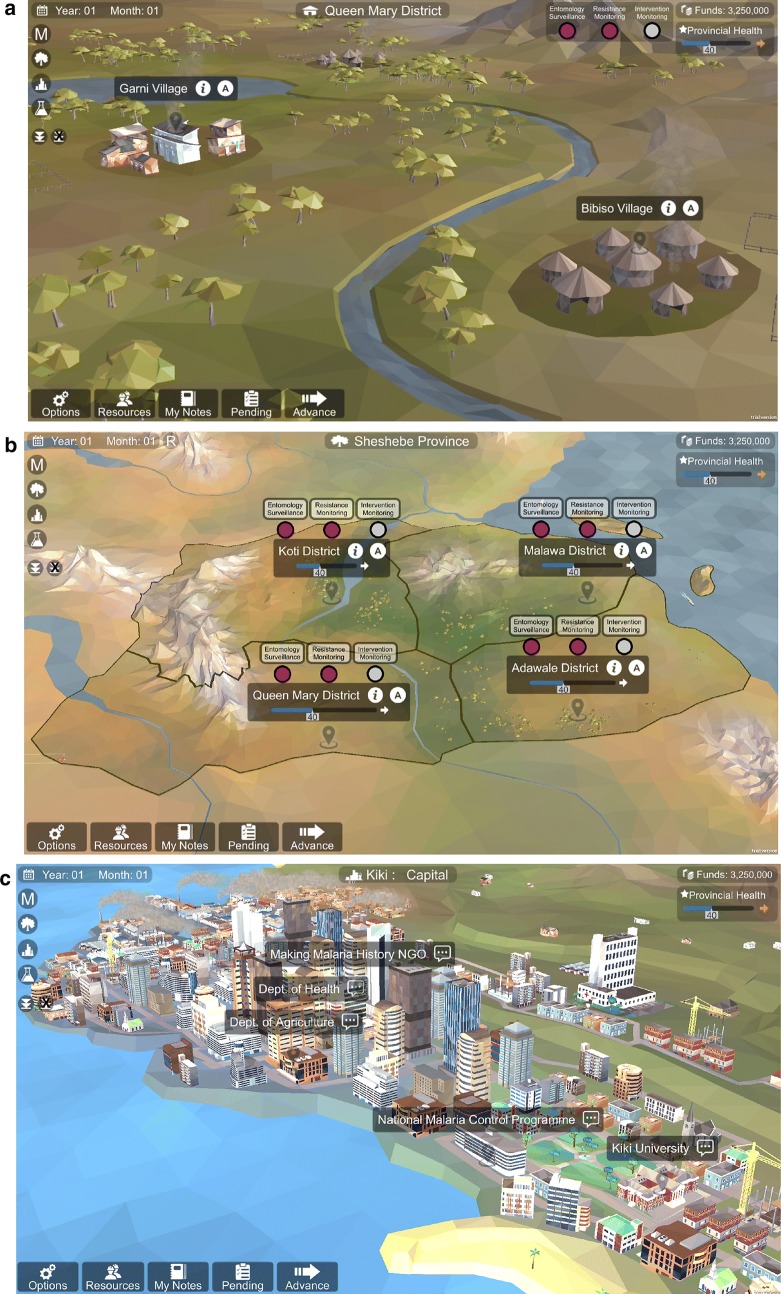
The three different map levels of ResistanceSim. **a** Shows the district level, which allows players to perform actions in three locales per district. **b** Shows the province level, which allows players to perform actions in four districts. **c** Shows the national capital, which allows players to interact with stakeholders

ResistanceSim is a management simulation game that immerses players in a fictional sub-Saharan African country. The player can interact with several environments (Fig. 1). At the province level, the player sees four districts. By clicking on one of the district labels, the game zooms into the district level, where the player can interact with several villages or towns. At the district level view, the player can rotate, pan, and zoom the camera to investigate their environment. At any time, the player can access the national capital, where they can interact with various stakeholders. Each geographical location in the game has different characteristics: the mosquito species present, their behaviour, their insecticide resistance profile, and the malaria transmission season and intensity all vary from place to place. There are a total of 12 locales that are defined as a village or town (three locales in four districts) that players can interact with.

The actions that the player can perform at each map level are different. At the district map level, the player can initiate entomological surveillance activities at any of the district locales (Fig. 1a). These activities involve collecting mosquitoes to monitor transmission intensity, vector behaviour, or insecticide resistance. Players can choose how they identify their mosquitoes (by morphology or PCR), which collection methods they use, and which assays they will use to characterize resistance. Any decisions made here will impact the data that is available to them later. At the province map level (Fig. 1b), the player can initiate interventions including the distribution of long-lasting insecticidal nets (LLINs) or IRS using various insecticides. In addition, they can perform community engagement, training, or intervention monitoring activities. At the national capital, the player can interact with stakeholders in various ways, including fundraising, sharing data, and participating in planning meetings (Fig. 1c). The player is in control of time, so they can queue up any number of actions across all geographical levels before advancing time. Once they do advance time, the game moves forward 1 month and any actions they have put in the queue will be completed. Each action is associated with a cost, and the appropriate amount of money will be deducted from the player’s budget as they perform actions.

**Fig. 2:**
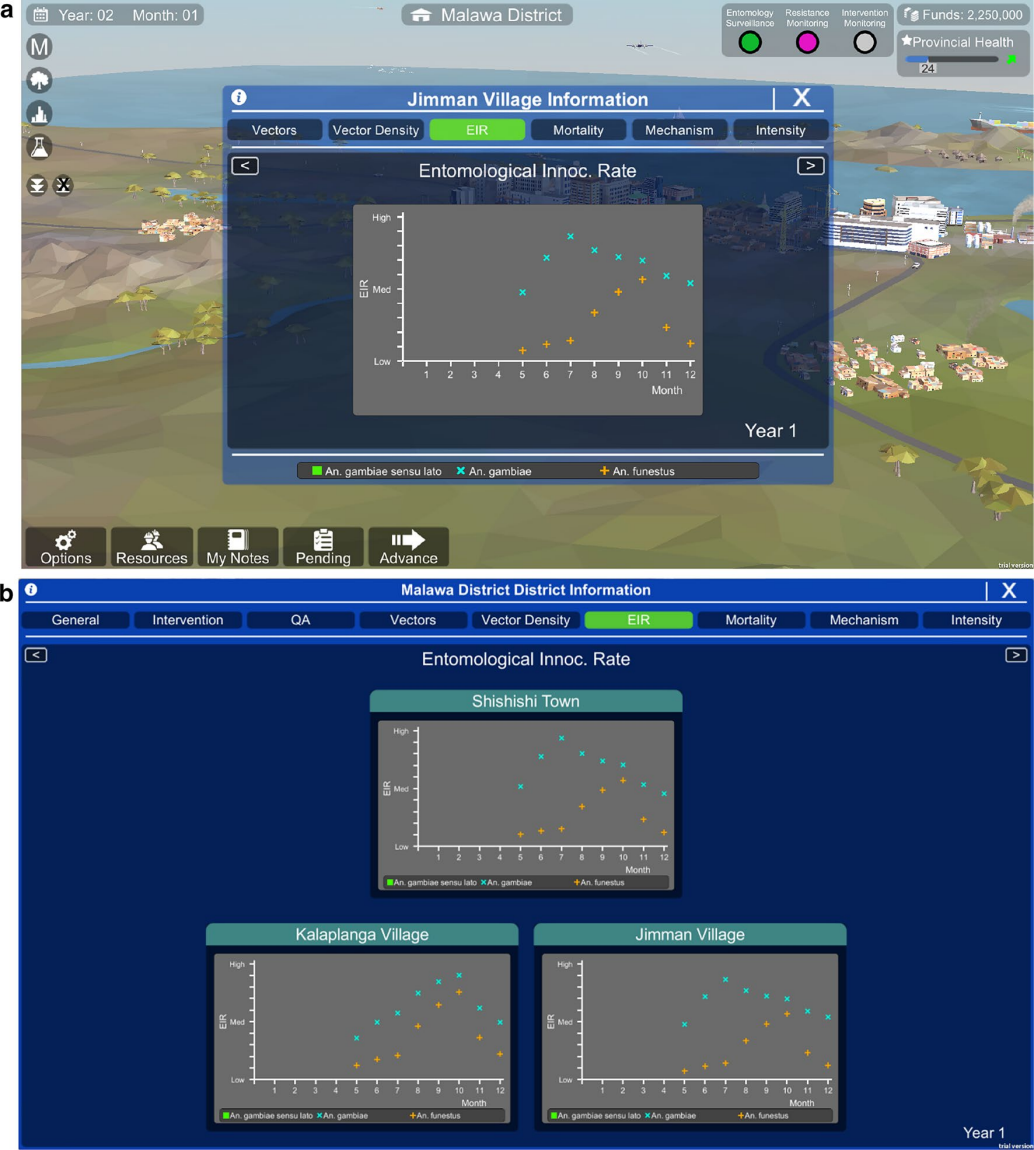
The data visualization components at the **a** district level and **b** province level. Players can collect and visualize data on vector species composition, behaviour, and density, malaria transmission, insecticide susceptibility, resistance intensity, resistance mechanisms, intervention quality, and residual efficacy

The player can view data that they collect from either the district level (Fig. 2a) or the province level (Fig. 2b). The data that appears in the data visualization screens is determined by what actions the player has performed. For example, if a player completes transmission monitoring activities in months 6–12 of year 1, but not months 1–5, they will only see the data for the second half of the year. The game model (described below) generates the underlying values for all the data visualization components. These values are influenced by the player’s decisions.

A very simple mathematical model (a few core lines of code) was developed to get the mosquito populations in ResistanceSim to react in realistic ways, in terms of both abundance and resistance levels, to player inputs. Therefore, if a player deploys an effective intervention, they will see the mosquito population go down. Conversely, if they deploy an intervention that the mosquito population is resistant to, they will see a less dramatic decrease. The model takes into account seasonal population fluctuations, frequency of resistance, intensity of resistance, resistance mechanisms, mosquito behaviour, intervention quality, intervention coverage, and community engagement, among other factors, in producing the outputs. In this model, a handful of parameters can be changed to generate various scenarios. The parameter values themselves are stored in an editable spreadsheet in the cloud, which allows the behaviour of the game to be changed without the game code itself being modified. The game model, which comprises approximately 20 lines of code, was originally written in R and can be found here: https://github.com/AndySouth/resistanceGame.

**Fig. 3:**
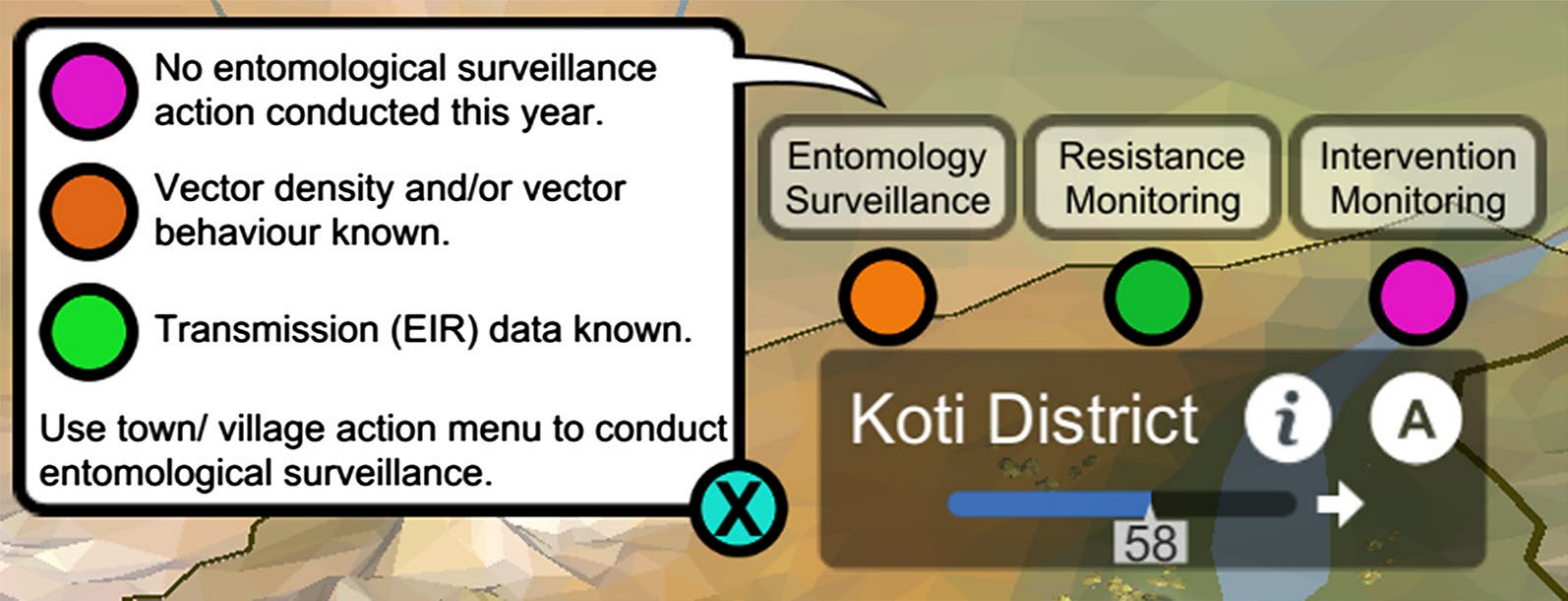
A simple stoplight visual to indicate whether the player has collected the recommended types of data. Clicking on the lights reveal hints for changing the colour of the light (shown on left)

**Fig. 4:**
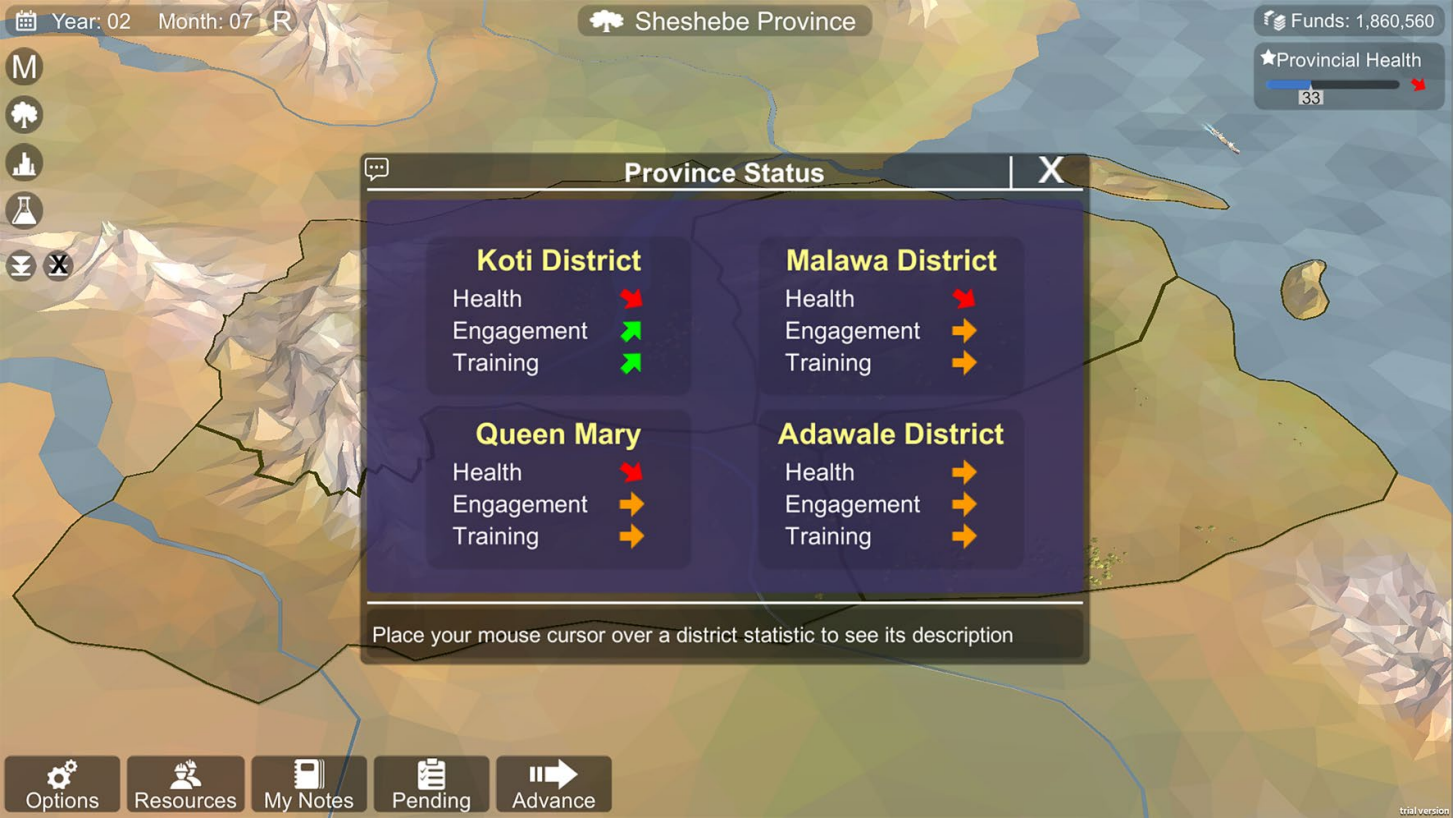
The feedback window that appears every time the player chooses to advance time. It gives a quick snapshot of how health, community engagement, and training levels are changing in each district

ResistanceSim includes several indicators of player progress so that the user understands how they are doing. First, there are a series of stoplight symbols above each of the district labels in the province map view (Fig. 3). These icons can either be pink, amber, or green, and provide a quick indication of whether the player has collected the recommended type of data in that district. Second, there are the district and province health bars. These bars indicate the relative health of that particular district or the province as a whole. The value displayed in these bars is directly related to the transmission, and therefore provides an indication of how well vector control is working. Lastly, after each advance of time, the player is presented with a summary of the training, community engagement, and health levels of each district, as well as an indication of whether each of these levels is going up or down (Fig. 4).

### Roadmap

**Fig. 5:**
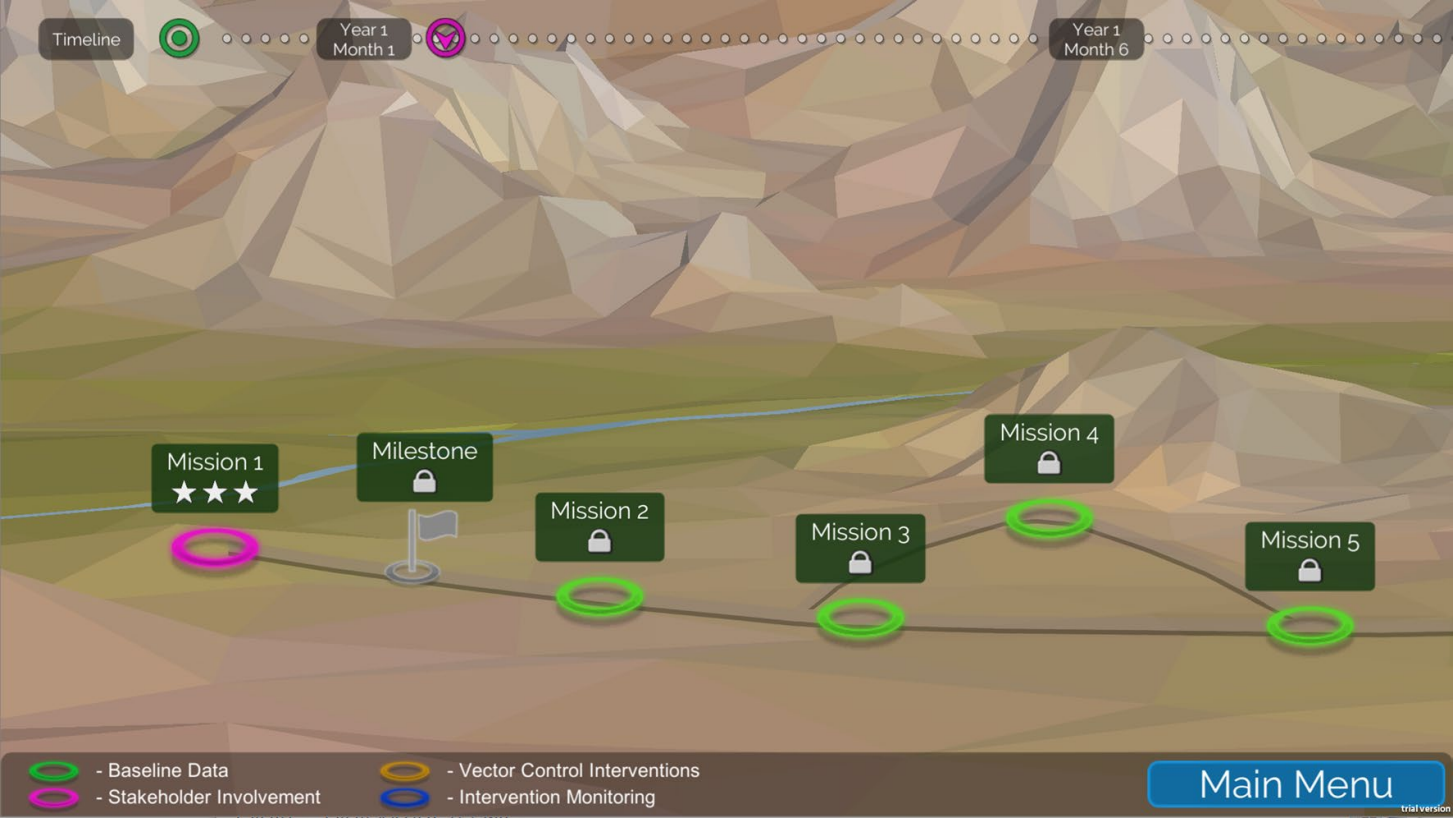
The Roadmap is a series of missions designed to provide structure to the simulation. The player starts with missions on stakeholder engagement and baseline data collection (shown in figure), and continues to play missions related to selecting interventions and monitoring the impact of those interventions

To provide the player with some direction as they are first learning how the simulation works, they can play through the Roadmap (Fig. 5). The Roadmap consists of a series of missions, each with its own goal, learning objectives, and decisions that need to be made. The missions follow a logical progression: engaging with stakeholders, collecting baseline data, followed by missions describing the data visualization components and how to interpret that data, and finally some missions on how to deploy interventions. The Roadmap continues so that players can then monitor their intervention, evaluate the data after the first year, and plan for another intervention the following year.

**Fig. 6:**
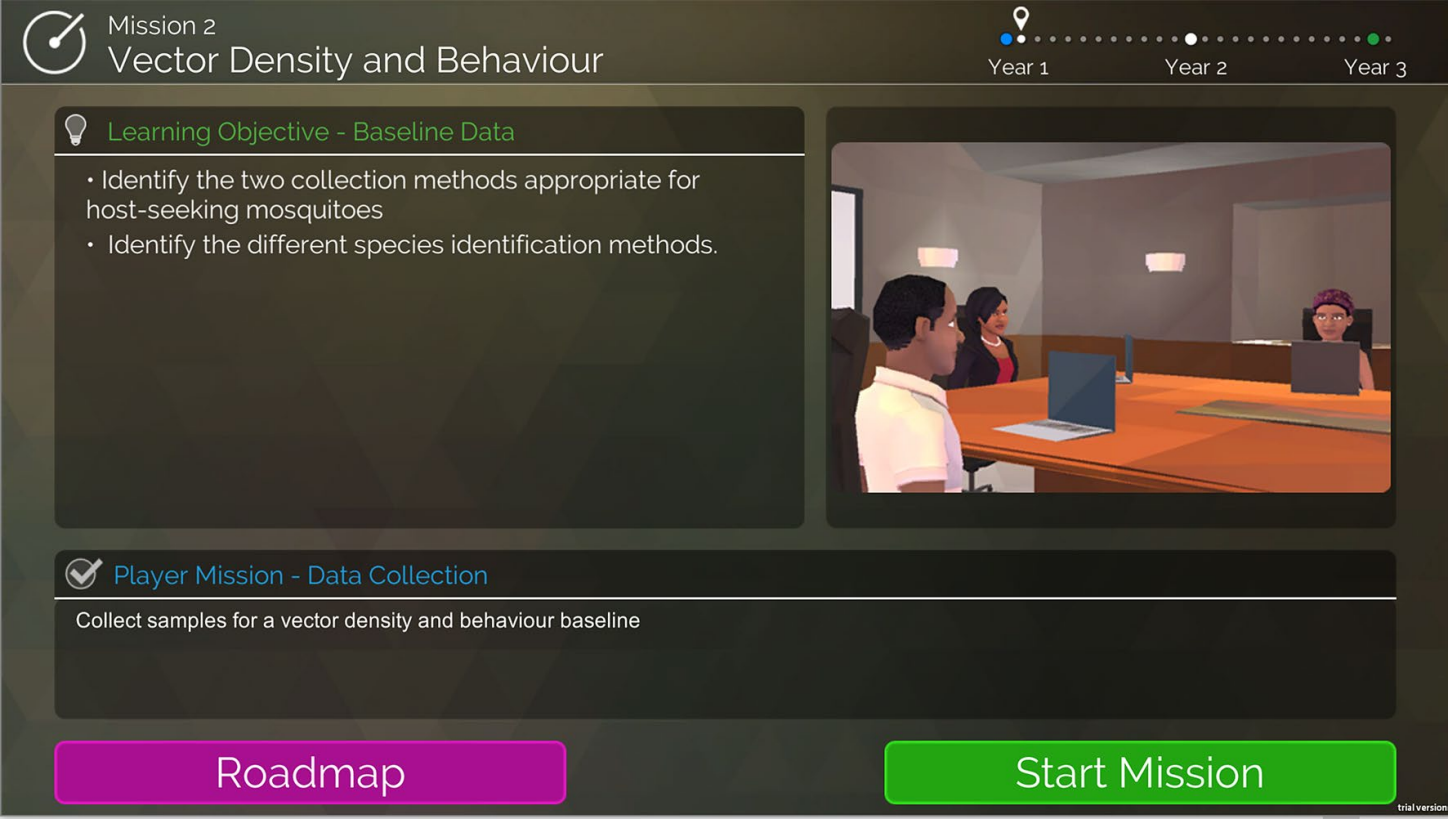
The mission start screen indicates to the player the learning objectives for this particular mission, and what the goal of the mission is

**Fig. 7:**
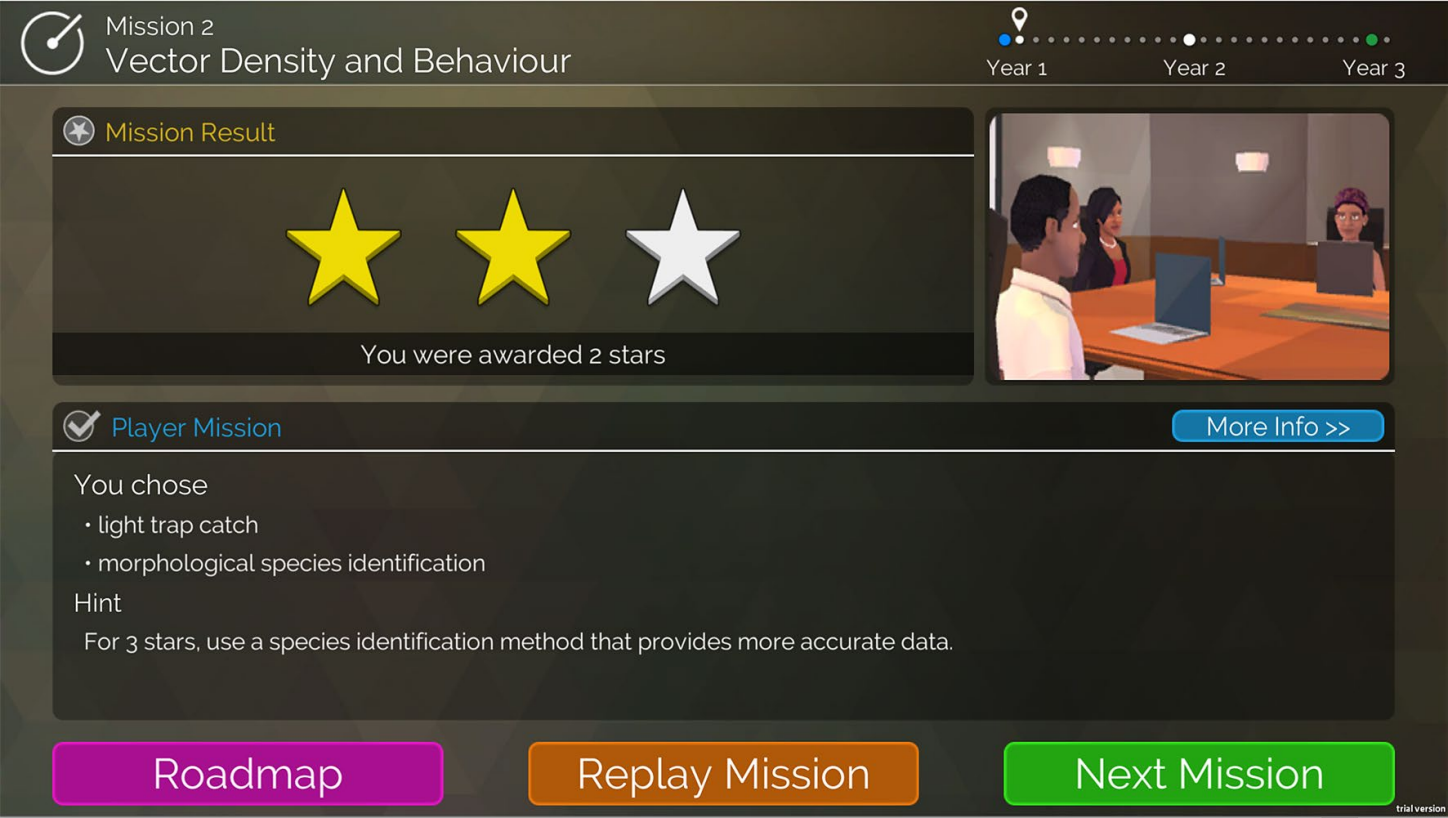
The mission feedback screen provides immediate feedback on the player’s decisions in the mission, assigning an overall star-rating for all the decisions that were made. It also provides hints one how to improve the star-rating. Clicking on “More Info” will provide the player with additional in-depth feedback on each of the decisions they made during the level, indicating why the decision was good or bad

Each mission begins with a start screen that describes what the goal of the mission is and what the player is expected to learn (Fig. 6). Once the player presses “Start Mission”, they are guided through the various steps required to complete their goal. Depending on the decisions they make during the mission, players can receive various star-ratings on the feedback screen upon mission completion (Fig. 7), with good decisions earning players more stars. The feedback screen also describes why players received their particular star-rating, and provides hints on how to get more stars.

### IRM course

The Roadmap and the open simulation described above were incorporated into a gaming-enhanced insecticide resistance management training course. This course lasts between 2 and 3 days, depending how many modules the course facilitator wants to include. In its most condensed form, the course begins on the first day with a series of mini lectures interspersed with short bursts of gameplay in the Roadmap. This allows students of all backgrounds to begin playing the game with the same foundational knowledge. Mini lecture topics include: mosquito collection methods, vector control tools, insecticide resistance and how to measure it, intervention monitoring strategies, etc. The mini lecture on a particular topic is given just before students play through the corresponding mission, so they have the opportunity to apply their learning immediately.

The second day comprises group work and gameplay in the open simulation. Students are given one of several IRM strategies to employ in the open simulation. They are then given the opportunity to implement this strategy for several hours. At the end, each student or group presents the results of their strategy to the rest of the class, so that all students can benefit from each other’s experience.

### Platform

ResistanceSim was produced using the Unity game engine for use on Windows and Mac-based PCs, as well as android tablets. The complexity of the user interface prevented the adaptation of the game for smartphones due to the average size of screens. It can be used with or without an internet connection.

### Development process

**Fig. 8:**
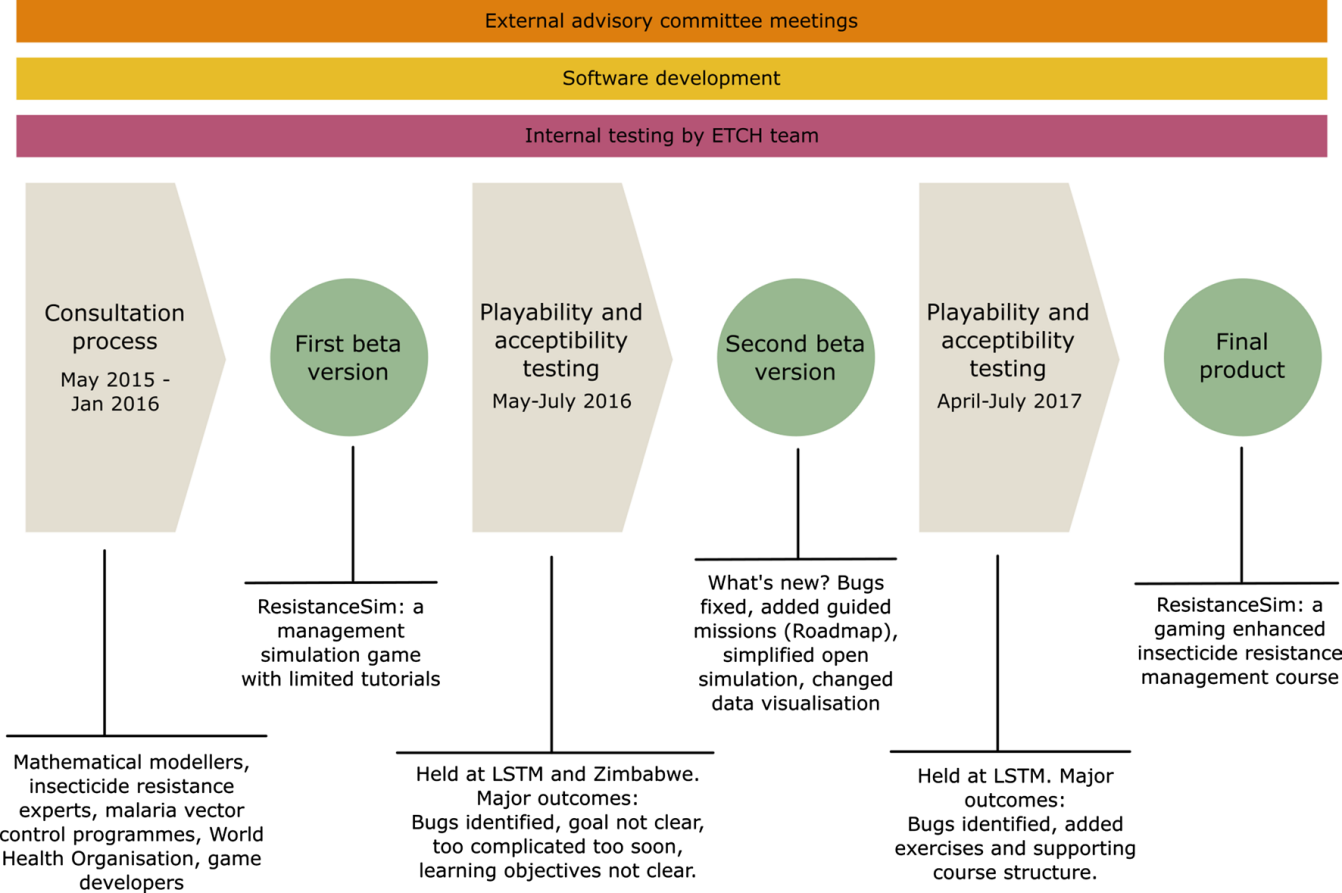
The processes involved in the development of ResistanceSim. Ongoing activities are indicated in the three boxes at the top

The development process for ResistanceSim continued for just over 2 years from May 2015 to September 2017 (Fig. 8). It generally followed the ADDIE instructional design framework, which organizes the development of instructional materials into analysis, design, development, implementation, and evaluation procedures [13]. This manuscript highlights the analysis, design, and development processes.

The first step involved convening stakeholders to analyse the need for such a tool, define the learning objectives and target audience, clarify the role of mathematical models in the game, and identify delivery strategies. Software developers were then engaged and learning objectives were mapped to game elements in a living game design document. Development sprints were interspersed with frequent user testing and external advisory committee meetings. These processes are detailed below.

### Stakeholder workshops

Two workshops were held early in the ResistanceSim development process. The first was held over 2 days in May 2015 with the primary aims to discuss and determine (1) the learning objectives that would be incorporated into the game specification, (2) the value and potential use of current disease control mathematical models to support learning objectives, and (3) game design and scenario options to best support the learning objectives. Participants at this workshop included representatives from malaria control programmes in sub-Saharan Africa and Southeast Asia, mathematical modellers, potential funding partners, members of the Engaging Tools for Communication in Health (ETCH) team at the Liverpool School of Tropical Medicine (LSTM), and the WHO.

The objectives were achieved through a guided brainstorming session using a modified Charrette procedure [14], followed by group discussions. Prior to the workshop, the organizers identified four major categories of activities related to IRM where vector control programmes currently face challenges: planning and implementation of IRM strategies, resistance monitoring, current and new tools (for surveillance, control, quality assurance, etc.), and the biology of resistance. There was also an “other” topic to capture challenges that did not fit easily into a single category. Workshop participants were placed in groups of 4–5 individuals, and each group spent 10 min brainstorming challenges faced by vector control programmes related to a single topic. They rotated until all groups visited all topics. Challenges were summarized by the workshop leaders and re-phrased into potential learning objectives. The mathematical modeller participants provided their expert opinion on whether/how each learning objective could be supported by the use of existing mathematical models. These discussions allowed the workshop organizers to produce a living document that defined the game’s learning objectives and the role of mathematical models in supporting these objectives.

The second workshop was held over 2 days in January 2016. The objective of this workshop was to define the preferred rollout strategy for the game, including how to make the game available and how it should be used. Participants in this workshop included representatives from malaria control programmes in sub-Saharan Africa, potential funding partners, the ETCH team, WHO Global Malaria Programme (GMP), and Abt Associates, the implementers of the President’s Malaria Initiative (PMI) Africa Indoor Residual Spray (AIRS) project. Opinions of the workshop participants on various aspects of the rollout strategy were gathered through interactive polling (Turning Technologies).

### Advisory committee meetings

Quarterly advisory meetings were held with an external panel. Panel members had expertise in insecticide resistance, pedagogy, and public health. The advisory committee provided direction across several different aspects of the development project, including the technical accuracy of ResistanceSim, the teaching strategies embedded in the tool, and the methods used to evaluate it. They also provided recommendations on synergies with existing research or vector control implementation projects.

### Playability testing

Routine testing was conducted throughout the development of ResistanceSim by the ETCH team. Playability testing with external users was performed four times coinciding with major development milestones. The primary objective of these testing sessions was to identify bugs and usability issues. However, if the testers were members of the target audience, a secondary objective was to assess acceptability as a learning tool.

In May 2016, 26 users were recruited from LSTM and stakeholder organizations to test the first beta version of ResistanceSim. This version had all the required functionality but had not been tested to ensure it was free of defects. Users were given a copy of the software with instructions on how to install it on their personal laptops. They were also given a structured spreadsheet that allowed them to capture usability issues as they were playing, and were asked to complete a short survey rating their experience playing the game. They answered questions about their engagement, the ease in which they learned how to play the game, and how easy it was to understand the various components. Users were then allowed to play the game in their own time over the course of 8 days, and their responses were collected afterwards via email. All bugs identified during this beta testing were fixed prior to further user testing.

The second major testing session occurred in Zimbabwe in July 2016. During this time, the AIRS project was conducting a regional entomological training session. It included 30 participants representing malaria vector control programmes from 11 countries in sub-Saharan Africa. For this session, the game was tested on the final day of the week-long training course. Participants were asked to complete a short pre-game survey to capture demographic information and awareness of IRM training resources. The survey also included Likert-scale questions to evaluate participants’ perceptions of demand for IRM training tools, of their own IRM knowledge, and of games and people who play games. It also included an open-answer question asking them to describe the steps involved in IRM. Then, participants were given a brief introduction to the game and could play on their personal laptops for approximately 3 h while a facilitator circulated around the room to answer any questions. After the play session, participants were placed in groups and provided with discussion questions in one of three topics: positive aspects of the game, barriers to a positive user experience, or barriers to sustainable implementation. After 20 min, the groups rotated in a Charrette procedure (described above) so that all groups contributed to all topics. At the end of this workshop, the facilitator led a discussion about each topic and asked groups to explain or expand on certain aspects. Audio from the discussion was recorded. Participants were asked to complete a post-game survey that included many of the same questions as the pre-game survey, but in addition asked them for their perceptions on individual game elements, as well as the value of the game as a whole. This research was approved by institutional review boards at the LSTM (Protocol 16-016) and the Medical Research Council of Zimbabwe (Protocol MRCZ/E/140).

Results from the Likert-scale survey were summarized with standard statistical measures of mean and standard error. Any comparisons between pre- and post-questionnaires were made using paired t-tests. The audio from the workshop was transcribed and analysed inductively. Illustrative quotes for each theme were documented.

Results from both testing sessions described above were fed back into another large development sprint which lasted for approximately 9 months. Major changes were made during this time to improve usability of the tool. The third playability testing session was held at LSTM in April of 2017, and included six users purposefully selected with expertise in education or operational vector control. These users were given a brief introduction to the tool, and were allowed to play through the game for 3 h, documenting any bugs or usability issues in a similar format to the first testing session. Pedagogical feedback on the delivery of the tool was particularly useful at this time, and was used to shape the development of a more comprehensive facilitated session. This facilitated session, which included gameplay, directed activities using the game, group work, and mini lectures was finally tested with a group of 20 individuals at LSTM in July 2017. The users included individuals well-versed with vector control and insecticide resistance, as well as those less familiar in order to gauge the response of a diverse audience. Bugs and usability issues were documented in a similar manner.

## Results

### Refining learning objectives and rollout

The original list of learning objectives generated from the first workshop included 21 items across the topics of vectors, resistance, disease epidemiology, chemical-based interventions, intervention monitoring and impact evaluation, finances, stakeholders, and unforeseen challenges. Over the course of designing, developing and testing the game, these learning objectives were further refined (Table 1). It was also recognized that certain learning objectives may take longer to achieve through gameplay than others, such as evaluating the cost-effectiveness of various intervention strategies.

**Table 1:** Complete list of learning objectives addressed in ResistanceSim

Topic	Learning objective
Stakeholders	Identify which stakeholders to involve in insecticide resistance management planning
Vectors	Compare the data obtained from various mosquito collection methods
	Compare the data obtained from different species identification methods
	Identify which collection method is required to determine transmission intensity
	Explain why it is important to use consistent collection sites
	Explain how vector bionomics influence intervention choices
Resistance	Describe the process of generating insecticide susceptibility data
	Identify the collection and test methods available to determine insecticide susceptibility, resistance intensity, and resistance mechanisms
	Describe the data required to construct a resistance profile
	Explain the importance of species identification in constructing a resistance profile and interpreting resistance data
	Illustrate the effect of continuously using insecticides with one mode of action
	Evaluate the different insecticide resistance management strategies available
	Apply this evaluation to make an appropriate resistance management plan
Evidence-based decisions	Explain why it is important to look at data before making an intervention decision
	Evaluate what insecticide class to use based on the resistance data
	Assess when to deploy an intervention based on vector density and transmission data
Intervention monitoring	Explain why it is important to use consistent methodology for routine monitoring
	Identify the information that different intervention monitoring tools provide
	Explain how quality assuring interventions contributes to insecticide resistance management
	Compare the information gathered from different monitoring tools
	Explain why it is important to monitor transmission
	Explain why it is important to monitor insecticide susceptibility, resistance intensity, and resistance mechanisms
	Demonstrate how to improve the quality and coverage of an intervention
Finances	Evaluate the cost-effectiveness of various intervention strategies

These learning objectives were first identified during stakeholder workshops, and further revised during the game development process

Workshop participants identified that malaria transmission models, including OpenMalaria and EMOD, were more detailed than necessary to support the learning objectives and at that time had little consideration of insecticide resistance. Even if they were thought suitable, it would be impossible to get these models to run in the background of the game due to a lack of computing power. Population genetic models to predict the evolution of insecticide resistance [15] also contain more detail than is necessary to support the learning objectives. Since ResistanceSim is designed to be a learning tool, and not a decision-support tool, it was decided that an extensive validated model was not needed. All that was needed was something that would generate outputs to the players within game scenarios that would support individual learning objectives. This, therefore, led to the development of the ResistanceSim game model (described above). In order to develop and test the model outputs, a web application was developed using the Shiny package (RStudio Inc.) to allow the development team to manipulate model parameters and test various scenarios quickly and easily. Simultaneously, the game developers transferred the code to C#, the language used by game development platform Unity, so that the mosquito populations in the game reacted as expected.

Participants in the rollout workshop felt that the game should be incorporated into existing IRM training activities, rather than being played individually or in a separate session. In addition, it was decided that playing the game as part of a facilitated session would have the most impact. To encourage the uptake of the tool, it was suggested that a comprehensive curriculum and course structure were created and distributed with the game itself. This would serve as a facilitator’s guide, and make it easier for country vector control programmes to adopt the tool.

#### Beta testing 1

**Fig. 9:**
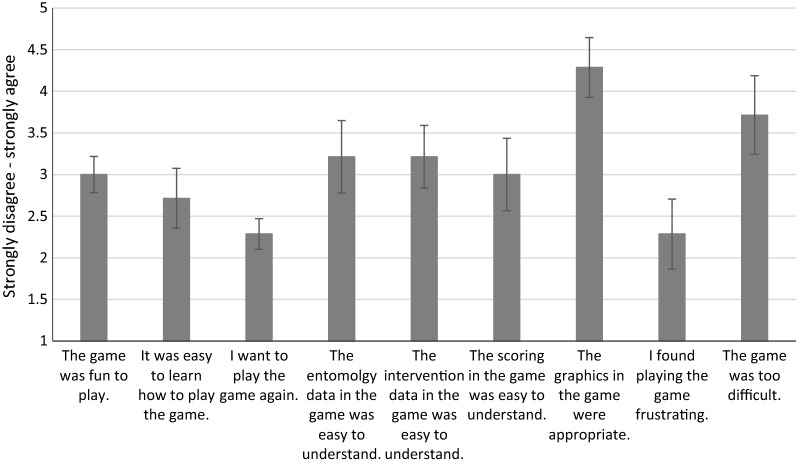
User perceptions (n = 8) of the first beta version of ResistanceSim. Error bars represent the standard error of the mean

Results from the first beta testing session held at the LSTM and remotely with other stakeholders produced a list of 32 bugs. Usability issues were numerous, and included confusion about the tutorial section, how data is presented in the game, and whether players’ decisions were good or bad and why. Players’ opinions of the game at this time were neutral, neither agreeing nor disagreeing with many of the survey questions (Fig. 9). After discussing these issues with the beta testers and amongst the ETCH team, a list of 79 change requests were produced to help address some of the issues with data visualization, the tutorial section, and player feedback. Prior to the next testing session in Zimbabwe, all bugs were fixed, and change requests were prioritized to focus on the clarity of the tutorial section and data visualization.

#### Beta testing 2

**Fig. 10:**
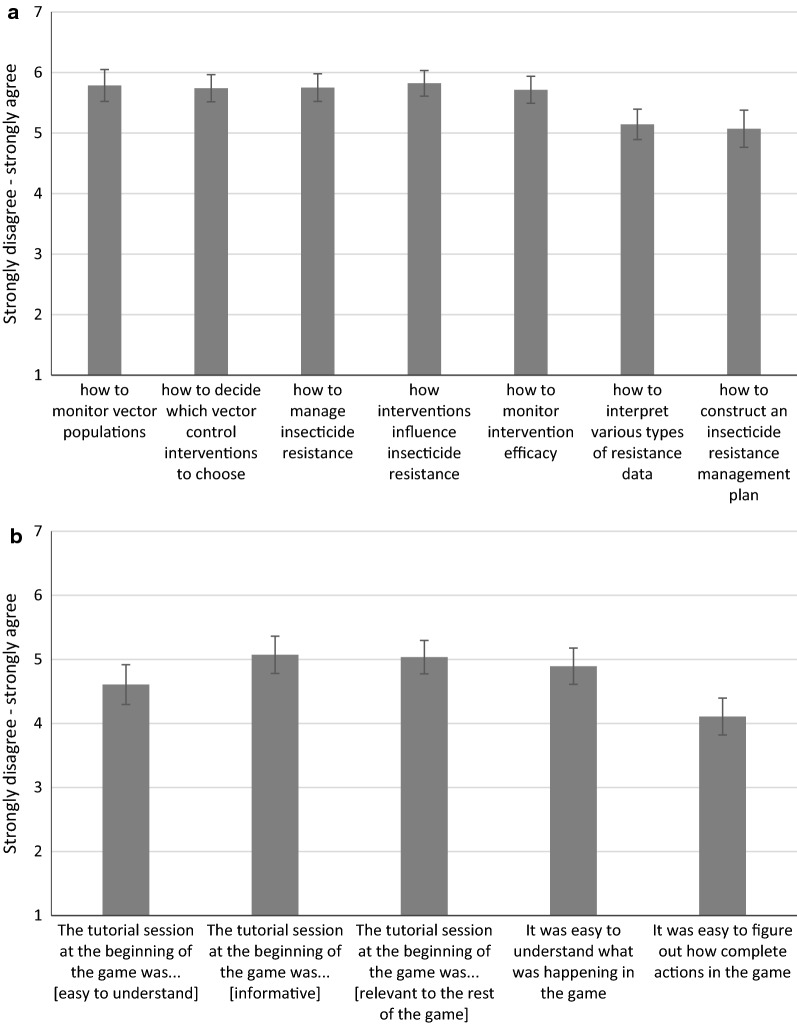
User perceptions (n = 28) during the second beta testing session in Zimbabwe of **a** the degree to which ResistanceSim improved their understanding of various topics and **b** the ease of use of the tutorial section. Error bars represent the standard error of the mean

User experiences in Zimbabwe were more positive than in the first testing session. Users generally felt that the game improved their understanding of various topics related to vector control (Fig. 10a), and that the data presented in the game was easy to understand. The tutorial section was still difficult for users to work through, and this was reflected in both the survey answers (Fig. 10b) as well as the progress that most people made during the test session—only 4 out of 30 participants were able to make it past the tutorial during the 3-h play session.

Feedback during the workshop discussion shed more light on the positive and negative aspects of the game. Players enjoyed the fact that their own actions in the game influenced the outcomes: “*We also like the interpretation of data where you could see the impact of IRS on vector density.”* They also expressed satisfaction in the complexity of the topics covered in the game: “*We liked how the game instructed you to start your activities at national level, then move to the province, to the district, down to the village … this makes you aware of the need to involve all levels in terms of implementation and planning.”* However, it also became clear that while complexity in the topics covered was desirable, complexity in the user interface was preventing users from interacting meaningfully with content: “*Rather than spend maybe an hour or 2* *h just cracking your head trying to work out how to navigate around and figure out how to play the game, I feel like you need to be able to jump in a lot more quickly.”* In addition, users expressed frustration in the way the instructions are presented: “*We are not engaging with the game. The actual reason (for this) is that the instructions are not clear.”* They were also disappointed by the lack of direction: “*I can see the provincial health bar going up and down, but there is no specific goal,” “As a player, you should be able to monitor independently how you are doing as far as your learning,”* “*It should have different levels.”*

Despite these difficulties, 90% of participants indicated that they need more support related to IRM, and they agreed that the tool would be a valuable addition to the training currently available for IRM and vector control (Fig. 10b).

All of the feedback from the LSTM and Zimbabwe testing sessions were consolidated and solutions were proposed to address most of the usability issues. The solutions fell in two categories: tutorial and interface improvements. The tutorial section was reworked replacing it with a guided, mission-oriented “Roadmap.” This guides players through the various aspects of the game itself, while slowly introducing the complexity of the content. The Roadmap provides immediate feedback on player decisions, so that they know what they are doing well and why. The second category of game changes involved simplifying the user interface of the open simulation while retaining the complexity of the content it covered. Changes in this category included reworking how data is displayed, removing extraneous aspects of the user interface, and providing regular updates to the player about how their decisions are impacting game outcomes. All of these changes were completed over a software development sprint lasting approximately 9 months.

### Usability testing

Results from usability testing in April 2017 indicated a vast improvement in the game. All participants (n = 6) felt that ResistanceSim would be a valuable addition to an IRM course. In contrast to the first beta testing session conducted in May 2016 (Fig. 9), all participants indicated that they wanted to play the game again. However, most also felt that in order to get the most out of the game, players needed to spend more time with it: *“…by the time you get through the missions (Roadmap), I felt then prepared to go into the game. But it’s almost like you need some thinking time…it requires time to get the most out of it.”* In addition, one participant who did not have a background in vector control found it difficult to understand what they were doing because of unfamiliarity with some of the terminology used. It was suggested that additional learning material be provided that allowed all users to start with the same level of knowledge. A total of 22 bugs and 14 usability issues were identified and documented in both the Roadmap and the open simulation. Most of the usability issues related to the transition between the Roadmap and the open simulation, where users are introduced to some new functionality that is not explicitly described. Feedback from this session resulted in two major developments. First, a series of short tutorial videos were created to ease the transition from the Roadmap to the open simulation. Second, a structured lesson plan and additional teaching resources were created (exercises, discussion topics, and slide sets) so that ResistanceSim was integrated into a facilitated course on IRM.

### Training course

The facilitated ResistanceSim training course was tested with 20 individuals at LSTM in July 2017. The course lasted from 0900 to 1600 h with time for breaks. Feedback was gathered through a simple open questionnaire that asked participants what they liked about the course and what could be improved. In general, participants were enthusiastic about the tool, and expressed satisfaction with the complimentary course material: “*I liked the linkage/balance between course instruction and activities,” “[the additional components] added considerable value to the ResistanceSim game itself.”* The value of the Roadmap was recognized as a way to slowly introduce complicated concepts: *“I liked the look and atmosphere of the application and the way that the structure built up as you got further into the modules and I started to make linkages and adopt reinforced behaviours* etc*.”* In addition, some suggestions were made to improve the exercises and group work that were completed as part of the course so that all participants can equally benefit from the ResistanceSim tool itself.

## Discussion

A ‘serious game’ was developed aimed at improving understanding of insecticide resistance management strategies among vector control programme personnel, with the ultimate goal of influencing decision-making processes. Over the course of 2 years, the game was evaluated for its validity through consultation with experts and external advisory boards, and frequent user testing focused on playability and perceived usefulness. The results from this work are promising, in that the final user-led product has been deemed a valuable potential addition to IRM training activities. As serious games have been shown to have positive impacts on knowledge and motivation [16], an important next step will be to evaluate ResistanceSim for its effect on knowledge acquisition, self-efficacy, and decision-making behaviours in vector control programmes that have used the game as a training tool.

Serious games have been used extensively in the health field, particularly aimed at training health professionals [12, 17] or changing behaviour of patients to improve their health outcomes [10, 11, 18]. However, to our knowledge, there are no serious games that target public health policy implementers, whose decisions have a massive impact on the health of many individuals. In addition, are only few examples of games being used in low and middle income countries or focused on diseases of poverty [19–23]. With computer use ubiquitous across multiple sectors in sub-Saharan Africa, and continuing to increase [24], this presents a significant opportunity to utilize technology as a capacity strengthening tool.

Previous literature reviews highlighted the necessity of iterative evaluation of instructional elements, gameplay mechanics, and user interface [25] when designing serious games. The results from this study reiterate this recommendation. Despite the early involvement of subject experts, game designers, and regular reviews from an external advisory committee, the first beta testing revealed that users simply did not enjoy playing the game. It was only after additional revisions and testing that a product was produced that struck the right balance between engagement and instruction that motivated users to keep playing.

The iterative nature of the development process also allowed the elucidation of potential implementation strategies, since users indicated that the game should be used as part of a structured course. This allowed us to test the game in this context during the final stage of development. The instructional resources are available for open use (at etch.lstmed.ac.uk), so that potential ResistanceSim course facilitators have guidance on the curriculum and structure of the course.

## Conclusions

In order to ensure the sustainability of public health insecticides, they must be used judiciously and intelligently. Strengthening the capacity of malaria vector control programmes to manage insecticide resistance is a critical component of this, but training resources are limited. ResistanceSim, developed here, is a management simulation game that immerses the player in a fictitious vector control programme, to fill this gap. Early and repeat testing with target users and involvement of stakeholders was vital in the development of the tool. This process has enabled us to improve user experience and provide a viable environment for learning. The potential for this serious game to be useful in training has been demonstrated, and its utility in operational settings is currently being tested.
